# Lipidomic Analysis of Archival Pathology Specimens Identifies Altered Lipid Signatures in Ovarian Clear Cell Carcinoma

**DOI:** 10.3390/metabo11090597

**Published:** 2021-09-03

**Authors:** Sartaj Ahmad Mir, Soon Boon Justin Wong, Kothandaraman Narasimhan, Chua W. L. Esther, Shanshan Ji, Bo Burla, Markus R. Wenk, David S. P. Tan, Anne K. Bendt

**Affiliations:** 1 Singapore Lipidomics Incubator, Life Sciences Institute, National University of Singapore, Singapore 117456, Singapore; esther.chua@nus.edu.sg (C.W.L.E.); lsijish@nus.edu.sg (S.J.); bo.burla@nus.edu.sg (B.B.); bchhead@nus.edu.sg (M.R.W.); anne.bendt@nus.edu.sg (A.K.B.); 2 Department of Biochemistry, Yong Loo Lin School of Medicine, National University of Singapore, Singapore 117596, Singapore; 3 Department of Pathology, National University Hospital, Singapore 119074, Singapore; 4 Singapore Institute for Clinical Sciences, A*STAR, 30 Medical Drive, Singapore 117609, Singapore; Kothandaraman_Narasimhan@sics.a-star.edu.sg; 5 National University Cancer Institute, National University Hospital, Singapore 119074, Singapore; david_sp_tan@nuhs.edu.sg; 6 Cancer Science Institute, National University of Singapore, Singapore 117599, Singapore

**Keywords:** ovarian cancer, lipid profiling, unsaturated fatty acids, desaturation, archival, formalin-fixed, clear cell

## Abstract

Cancer metabolism is associated with the enhanced lipogenesis required for rapid growth and proliferation. However, the magnitude of dysregulation of diverse lipid species still requires significant characterization, particularly in ovarian clear cell carcinoma (OCCC). Here, we have implemented a robust sample preparation workflow together with targeted LC-MS/MS to identify the lipidomic changes in formalin-fixed paraffin-embedded specimens from OCCC compared to tumor-free ovarian tissue. We quantitated 340 lipid species, representing 28 lipid classes. We observed differential regulation of diverse lipid species belonging to several glycerophospholipid classes and trihexosylceramide. A number of unsaturated lipid species were increased in OCCC, whereas saturated lipid species showed a decrease in OCCC compared to the controls. We also carried out total fatty acid analysis and observed an increase in the levels of several unsaturated fatty acids with a concomitant increase in the index of stearoyl-CoA desaturase (SCD) in OCCC. We confirmed the upregulation of SCD (the rate-limiting enzyme for the synthesis of monounsaturated fatty acids) by immunohistochemistry (IHC) assays. Hence, by carrying out a mass spectrometry analysis of archival tissue samples, we were able to provide insights into lipidomic alterations in OCCC.

## 1. Introduction

Ovarian clear cell carcinoma (OCCC) shows geographical differences in occurrence, with higher prevalence in Asia [1]. While being relatively understudied, OCCC is both clinically and molecularly distinct from the more common high-grade serous carcinoma of the ovary (HGSOC) [2]. Advanced OCCCs are more chemoresistant and portend a poorer prognosis compared with advanced HGSOC, and hence represent a significant clinical challenge for oncologists [3]. Better molecular characterization could potentially help in patient risk stratification and with the identification of novel targeted therapies. Recent studies have highlighted the importance of dysregulation of lipid metabolism in cancer [4,5]. Lipidomics studies provide a suitable platform to examine these molecular alterations [6], enabling the identification of lipid species involved in diverse biological processes with a potential to serve as candidate biomarkers for early diagnosis, prognosis, or monitoring of therapeutic response in cancer [7,8]. Several targeted and untargeted studies have documented the alterations in lipid species in various cancers [8,9,10]. Phospholipids and lysophospholipids are the most frequently reported dysregulated lipids [6]. Amongst sphingolipids, several sphingomyelin species [11,12], ceramides [13], GM_3_-gangliosides [14], and sulfatides [8] have been found to be altered. Ceramide and sphingosine-1-phosphate are involved in apoptosis and proliferation, respectively, making these potential therapeutic targets in cancer [15]. Similarly, autotaxin–lysophosphatidic acid signaling has been implicated in ovarian cancer [16,17,18]. Neutral lipids such as triacylglycerols, diacylglycerols, and cholesteryl esters have also been reported to be altered in cancer [19,20,21]. These studies highlight the importance of in-depth lipidomic analysis in cancers for elucidation of potential molecular signatures of carcinogenesis. However, there is currently a lack of lipidomics studies in OCCC. Formalin-fixed paraffin-embedded (FFPE) specimens can be stored easily and permit access to huge collections of previously archived patient material. Few metabolomics studies have measured metabolite level changes in cancers using FFPE specimens [22,23,24], but such studies are needed for the systematic characterization of lipid species in the cancer metabolome. Here, we describe a simple method that can be applied to FFPE specimens in order to extract lipids for downstream lipidomics applications. We have used this process to characterize lipidomic alterations in OCCC.

## 2. Results

### 2.1. Lipidomic Profiling of FFPE Tissue Samples

**Figure 1:**
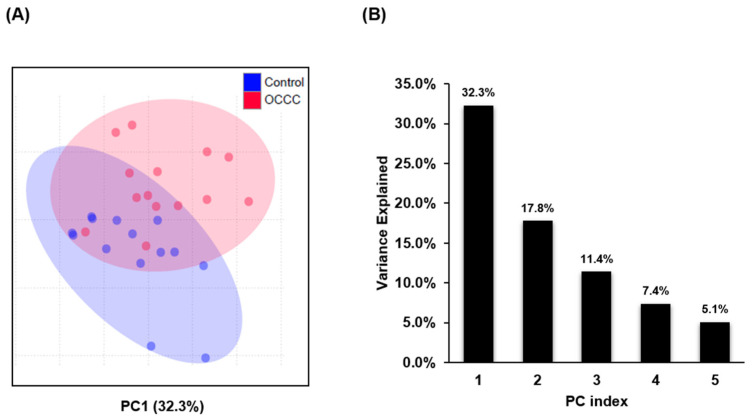
Overview of comparative lipidomic analysis of OCCC vs. control samples: (**A**) PCA analysis showing the group variance, PC1 explained 32.3% of variance, circles represent 95% confidence regions. (**B**) Individual variance contributed by the top 5 principal components.

Targeted LC-MS/MS analysis of lipid extracts from OCCC (*n* = 14) and control samples (tumor-free ovarian tissue, *n* = 14) resulted in the quantitation of 340 lipid species representing 28 lipid classes. Study subject details are provided in Table S1. LC-MS/MS methodology details, including MRM transitions and the corresponding internal standards, are provided in Table S2. Principal component analysis (PCA) showed separation between the two sample groups (OCCC vs. control tissue) and the variance was evident from PC1 (32.3%), as shown in Figure 1A. The top five principal components with individual contribution to the total variance are represented in Figure 1B.

### 2.2. Differential Expression of Lipid Species in OCCC

**Figure 2:**
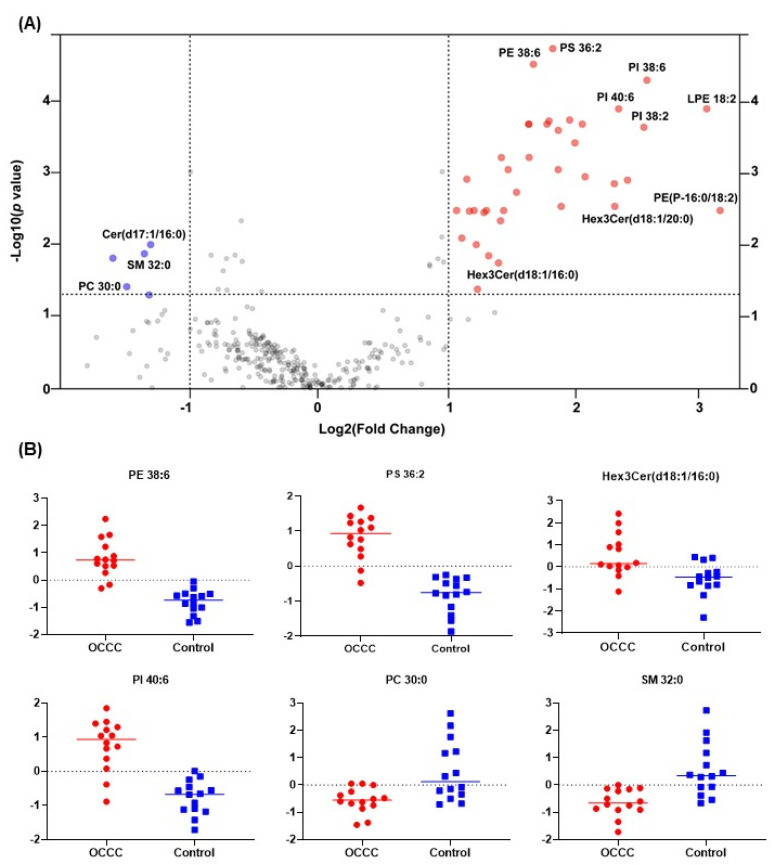
(**A**) Volcano plot showing lipid species that are significantly increased (red) or decreased (blue) in OCCC compared to control, *p* < 0.05 (FDR adjusted). (**B**) Individual dot plots of representative lipid species from several lipid classes showing differential expression in OCCC compared to control.

Having observed a noticeable separation between cancer and control samples based on lipid profiles, we carried out a further analysis to characterize the expression of different lipid species. For this, a univariate analysis was carried out to calculate fold change in lipid concentration (OCCC as compared to control) (Table S3) and two-sample *t*-tests (Table S4) were implemented. Volcano plot analysis was carried out to identify the differential levels of lipid species with a cut-off of fold change (≥2) and *p*-value < 0.05 (FDR adjusted). We observed differential levels in 43 lipid species, with 38 species that were increased in OCCC compared to the controls, and 5 species that were decreased (Figure 2A). Differential levels of several representative lipid species are depicted in Figure 2B. The complete list of lipid species from this analysis is provided in Table S5. Lipids that showed an increase in concentration were primarily glycerophospholipid species belonging to the phosphatidylethanolamine (PE), lysophosphatidylethanolamine (LPE), phosphatidylinositol (PI), and phosphatidylserine (PS) classes. A few ether-linked phospholipid species belonging to alkenylphosphatidylethanolamine (PE-plasmalogens) were also elevated. At the same time, glycerophospholipid species with saturated fatty acids were either unchanged or decreased in OCCC. Complex sphingolipid species belonging to the trihexosylceramide (Hex3Cer) class were also upregulated, whereas sphingomyelin species with low carbon number and unsaturation (saturated) were downregulated. These results were also subjected to pathway analysis using the LIPEA (lipid pathway enrichment analysis) [25] online tool, and this showed that the glycerophospholipid metabolism pathway was the most significantly regulated, along with other lipid metabolism pathways, which are represented in  Table S8.

### 2.3. Unsaturated Lipid Species Are Elevated in OCCC

We observed that one of the defining features of the glycerophospholipid species with higher abundance in OCCC was the presence of one or more double bonds in the fatty acid moieties (Table S5). Apart from monounsaturated lipid species such as PE 32:1, PE 34:1, and PI 34:1, several polyunsaturated fatty acid-containing lipid species were also elevated in OCCC, including phosphatidylethanolamine species (PE 38:4, PE 38:6, PE 40:6) and phosphatidylinositol species (PI 38:4, PI 38:6, PI 40:6). The differentially expressed plasmalogen species also contained unsaturated fatty acids, such as linoleic acid. The relative distribution of different lipid species from phosphatidylcholine, phosphatidylethanolamine, phosphatidylinositol, and sphingomyelin classes are represented as a percentage of the total class levels in Figure S1.

**Figure 3:**
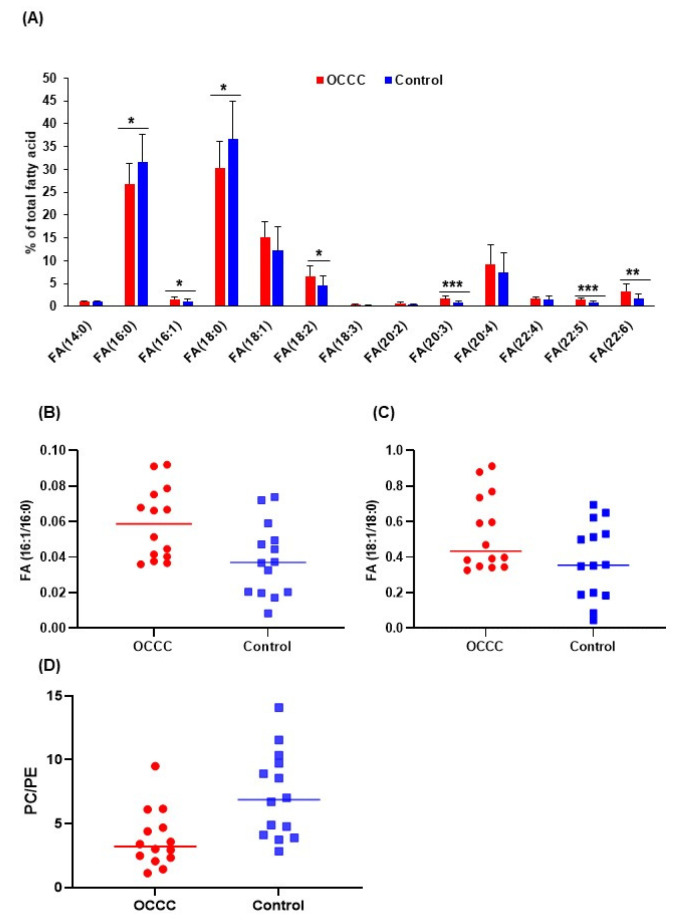
(**A**) Measured levels of individual fatty acids represented as a percentage of the total fatty acid content; saturated lipid species showed an overall decrease whereas unsaturated species were increased in OCCC compared to controls (* *p* < 0.05, ** *p* < 0.01, *** *p* < 0.001). Ratios of fatty acid (**B**) 16:1 to 16:0 (*p*-value = 0.01) and (**C**) 18:1 to 18:0 (*p*-value = 0.06), as measures of desaturation index, showed an overall increase in OCCC. (**D**) Ratio of total levels of phosphatidylcholine to phosphatidylethanolamine (*p*-value = 0.004) showed a decrease in OCCC.

The fatty acid analysis showed a substantial increase in monounsaturated (FA 16:1, FA 18:1) and polyunsaturated fatty acids (FA 20:3, FA 22:5, FA 22:6), whereas saturated fatty acids (FA 16:0, FA 18:0) were lower in concentration (Figure 3A) in OCCC compared to the control (Table S6). We also observed an increase in the ratio of FA 16:1 to FA 16:0 (Figure 3B), and FA 18:1 to FA 18:0 (Figure 3C), indicating an increase in fatty acid desaturation in OCCC. Apart from an increase in total levels of unsaturated fatty acid, we also observed a decrease in total PC-to-PE ratio in OCCC (Figure 3D).

### 2.4. Stearoyl-CoA Desaturase (SCD) Expression in OCCC and Control Tissue

**Figure 4:**
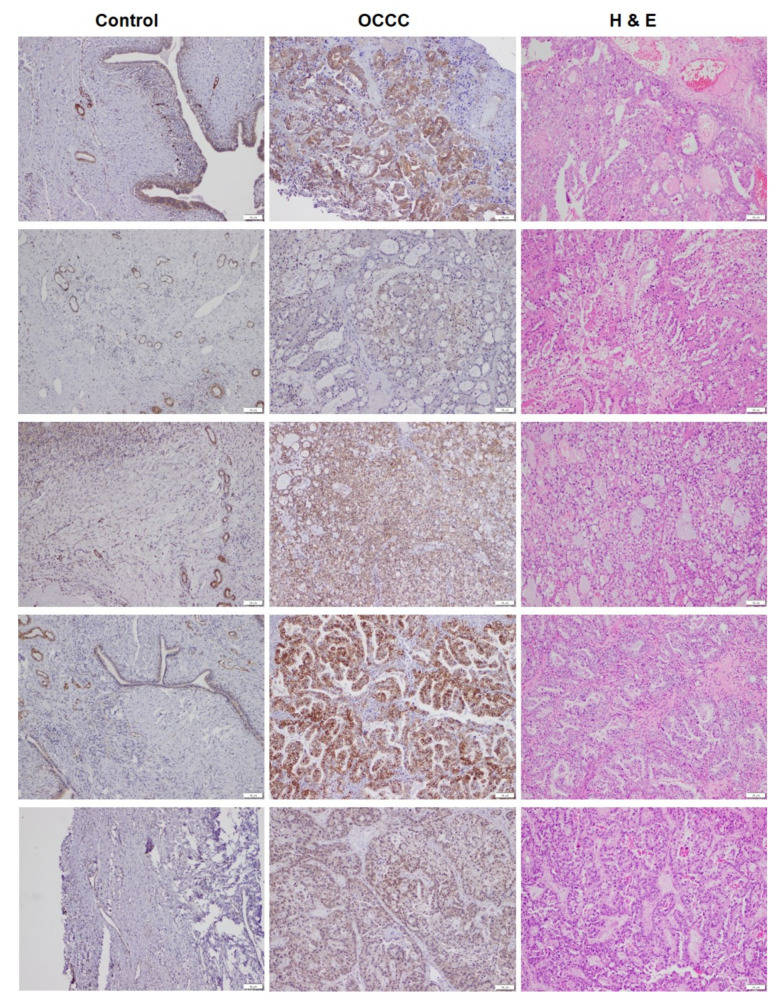
Immunohistochemical staining of SCD was carried out on tissue sections from OCCC and corresponding control ovarian tissue (*n* = 5 each, all sections are depicted at the same optical magnification, scale bar = 50 µm). There was increased expression of SCD in OCCC.

To follow up on the observations that many unsaturated lipid species were increased in OCCC, we carried out an immunohistochemical (IHC) analysis of SCD, the rate-limiting enzyme responsible for introducing the unsaturation in fatty acids. For this analysis, OCCC specimens from different patients, along with paired uninvolved contralateral ovarian tissue (*n* = 5) from the respective patients, were selected on the basis of tissue availability and examined by immunohistochemistry. Based on the IHC scores, SCD showed increased abundance in all five OCCC cases, with heterogeneous protein expression in the individual tumors (Figure 4). The IHC scores for these five OCCC patients are provided in Table S7.

## 3. Discussion

**Figure 5:**
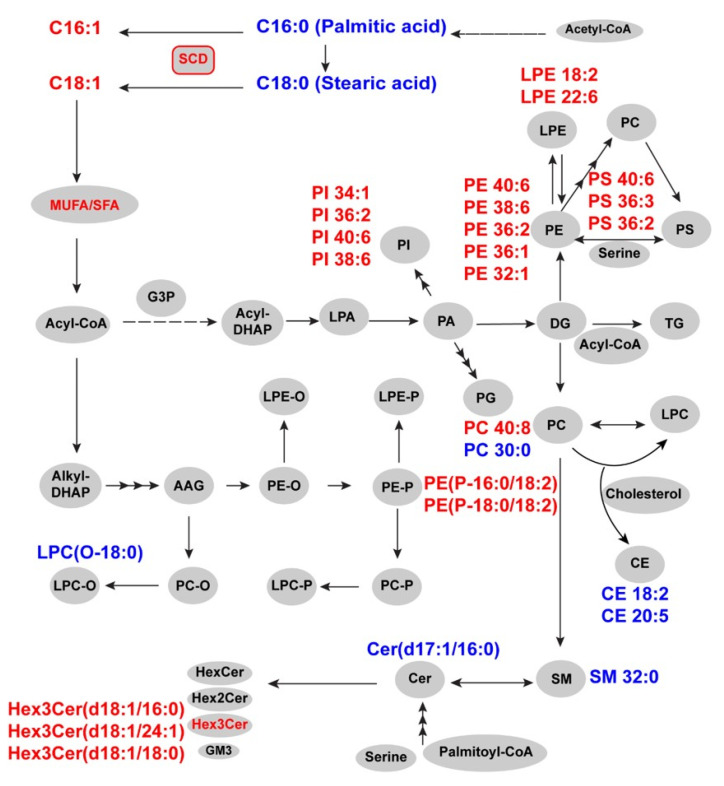
Mapping of molecular alterations in OCCC onto lipid metabolism pathways.

Alterations in cancer metabolism have been associated with tumor growth, metastasis, resistance to therapy, and survival of cancer stem cells [6,7,8]. Novel information about the role of lipids in cancer is now emerging [9,10]. Here, we have carried out a lipidomic analysis of FFPE specimens from OCCC and tumor-free ovarian tissue samples (*n* = 14). Out of the 340 lipid species profiled in this study, 43 (12.6%) showed significant changes in abundance in OCCC compared to the control tissue. The lipid species with increased concentration can be classified into glycerophospholipids (such as phosphatidylethanolamine, phosphatidylinositol, and phosphatidylserine), lysoglycerophospholipids (such as lysophosphatidylethanolamine), alkenylphosphatidylethanolamine (PE-plasmalogens), and complex sphingolipids (such as trihexosylcermaides). Amongst the lipid species with decreased concentrations were saturated fatty acids containing phosphatidylcholine and sphingomyelin species. Glycosphingolipids such as trihexosylceramides (including globotriaosylceramide, Gb3) were upregulated in OCCC. These Gb3 lipids are involved in the metastasis and aggressiveness of cancer [14,26,27,28]. The general trend in neutral lipids, such as diacylglycerol, triacylglycerol, and cholesterylesters, was either no change or a marginal decrease (CE 18:2, CE 20:5) in OCCC. Glycerophospholipids along with sphingomyelin are the main components of plasma membranes and constitute the bulk of lipids in tissue [29]. Apart from the structural role of these lipids, phospholipids contribute to the formation of other lipids and bioactive species such as lysolipids, which are generated by phospholipases [30]. Due to their central role in the cellular architecture, cancer cells may differentially regulate phospholipid metabolism to support their uncontrolled cell growth and division. The illustrations of the distribution of lipid species within the class highlight the fine tuning of differential levels of lipid species in cancer depending on their unsaturation and fatty acid composition (Figure S1). Further, plasmalogens contain vinyl ethyl bond at *sn1* position and have been implicated in fighting oxidative stress at the cellular level. Plasmalogens that contain polyunsaturated fatty acids (PUFA) have previously been shown to be involved in resistance to cell death in cancers [31]. In this study, we observed an upregulation of PE-plasmalogen species that contain linoleic acid. Further studies could address whether these lipid species play a role in cancer metabolism that is distinct from PUFA-containing species of plasmalogens. These species, along with other unsaturated phospholipid species such as PE 32:1, PE 34:1, PI 34:1, and PS 36:1, represent a common signature of enhanced fatty acid desaturation in OCCC. Our total fatty acid analysis further illustrated the overall increase in both monounsaturated and polyunsaturated lipid species in OCCC. Apart from decreased levels of saturated fatty acid-containing lipid species, we also observed a decrease in total levels of FA 16:0 and FA 18:0, two of the major fatty acids by composition, in OCCC. Further, by using the ratios of FA 16:1 to FA 16:0 and FA 18:1 to FA 18:0, we observed an increase in the desaturation index. Increase in fatty acid desaturation may have an impact on cancer cell architecture and increase membrane fluidity [32]. We estimated the ratio of total phosphatidylcholine to phosphatidylethanolamine [33] and observed a decrease in OCCC. PC and PE are the most abundant phospholipids in mammalian cell membranes, and any changes in the PC/PE ratio may influence membrane organization, making it more rigid or fluid [34]. Apart from the CDP-choline pathway, PC is formed from methylation of PE by phosphatidylethanolamine *N*-methyltransferase (PEMT) [30], and the decrease in PC/PE ratio indicates a change in membrane fluidity in OCCC [35]. These data suggest disruptions in lipid bilayer organization that may facilitate proliferation and metastasis [36]. Based on our observation that several unsaturated lipid species were increased in OCCC, we carried out IHC to determine protein expression of SCD in OCCC. We observed an overall increase in SCD levels in OCCC. Fatty acid desaturation represents a critical step in lipogenesis and SCD is a rate limiting enzyme that converts saturated fatty acids to monounsaturated fatty acids. A number of studies have demonstrated that de novo lipogenesis is enhanced in cancer [37,38], and SCD-dependent desaturation of the fatty acids is a critical event in this process [39,40]. Studies have elucidated the role of SCD in the maintenance of stemness, the regulation of apoptosis, and more recently in ferroptosis [41,42,43]. These studies have improved our understanding of the involvement of SCD in carcinogenesis and highlight its role as a potential therapeutic target. By integrating data from lipidomics, total fatty acid analysis, and IHC, we were able to provide a detailed view of the widespread perturbations in lipid species in OCCC. These changes were mapped onto metabolic pathways to provide an overview of lipid remodeling in OCCC (Figure 5). Pathway mapping of differentially expressed lipid species also reinforced the alterations in different lipid metabolism pathways (Table S8). To the best of our knowledge, this is the first attempt to characterize lipidomic changes in OCCC, and this information may improve our understanding and ability to exploit metabolic vulnerabilities in OCCC [44].

## 4. Materials and Methods

### 4.1. Reagents

MS grade acetonitrile, methanol, and 2-propanol were supplied by Fisher Scientific (Waltham, MA, USA). Formic acid, ammonium formate, and 1-butanol were supplied by Merck Pte Ltd. or Sigma-Aldrich Pte (Singapore). RIPA lysis and extraction buffer and monoclonal antibody for SCD1 (#MA5-27542) were supplied by Thermo Fisher Scientific. Lipid standards consisting of odd chain/non-physiological and deuterated species were purchased from different suppliers. Phosphatidylcholine (PC 13:0/13:0), phosphatidylethanolamine (PE 17:0/17:0), phosphatidylglycerol (PG 17:0/17:0), phosphatidylserine (PS 17:0/17:0), lysophosphatidylcholine (LPC 13:0), lysophosphatidylinositol (LPI 13:0), lysophosphatidylethanolamine (LPE 14:0), cholesterol (D7), ceramide-d7 (d18:1-d7/18:0), glucosyl (β) ceramide (d18:1/18:1), sulphatide d18:1/12:0, lactosyl(ß) ceramide d18:1/18:1, sphingosine-1-phosphate (Sph-1-P) 17:1, sphingosine (Sph) 17:1, sphingomyelin (SM d18:1/12:0), and dihydroceramide (d18:0/8:0) were supplied by Avanti Polar Lipids (Alabaster, AL, USA). Cholesteryl-2,2,3,4,4,6-d6 Octadecanoate was supplied by CDN isotopes (Quebec, Canada). Hexadecanoyl (16,16,16-D3)-L-Carnitine HCl salt was supplied by Larodan Chemicals (Solna, Sweden). Trihexosylceramide 17:0 was supplied by Matreya LLC (2178 High Tech Rd, State College, PA, USA). Diacylglycerol (DAG) 15:0 15:0 was supplied by Santa Cruz Biotech (Dallas, TX, USA) and triacylglycerol (TAG) 17:0 17:0 17:0 was supplied by Sigma Aldrich Pte. (Singapore). Oleic acid-d17 was supplied by Cayman Chemicals, Ann Arbor, Michigan, United States.

### 4.2. Methods

#### 4.2.1. Formalin-Fixed Paraffin-Embedded Tissue Sections

Ovarian tissue affected by OCCC, as well as contralateral ovary tissue that was resected during the same surgical operation and assessed by the reporting pathologist to be free of tumor, was used for this study. Control and tumor-bearing tissues were sampled and processed for histology in parallel, using identical protocols for formalin fixation and paraffin embedding. Tissues were fixed overnight at room temperature in phosphate-buffered 10% formaldehyde solution. Then, 3 mm-thick tissue sections were placed in cassettes, which were processed and embedded using an enclosed tissue processor (Epredia^TM^, ThermoFisher Scientific, Singapore). In brief, tissue sections were dehydrated using a graduated ethanol series, followed by a clearing sequence employing three changes of xylene solution, each lasting 30 min. Finally, the specimens were infiltrated with molten paraffin wax in another three-step procedure, each step lasting for 1 h, 1 h, and 2.5 h, respectively. All paraffin tissue blocks were subsequently stored at room temperature in a dedicated storage facility maintained by the Department of Pathology, National University Hospital, a laboratory accredited by the College of American Pathologists. For lipidomics analysis, 20 µm cores of FFPE tissue were obtained from OCCC and control tissue blocks, and the weight of each core was recorded.

#### 4.2.2. Sample Preparation

The FFPE cores were subjected to lipid extraction as described previously [22,23,24,45], with slight modifications. Briefly, single-phase lipid extraction was carried out by using methanol and 1-butanol in 1:1 (*v*/*v*), spiked with internal standards, as described in Table S2 [46,47,48]. In the initial step, 1 mL of extraction solvent was added into each tube to completely cover the FFPE core. The samples were then incubated at 70 °C for 1 h on a thermal mixer followed by 15 min on ice to congeal the wax. The samples were centrifuged at 14,000× *g* for 10 min and supernatants were collected in labelled tubes. The remaining portions were kept on ice again for 15 min, followed by centrifugation. The supernatants were collected and added into the respective collection tubes. These total lipid extracts were dried under vacuum and stored at −80 °C until LC-MS/MS analysis. The remaining pellets were washed with 1 mL of xylene, followed by centrifugation. The xylene was aspirated carefully without disturbing the pellet, and this process was repeated twice. The pellets were rehydrated by adding 1 mL of 100%, 70% and 50% ethanol in serial steps of centrifugation and aspiration. The recovered pellets were air dried and solubilized in RIPA lysis buffer for protein estimation using BCA assays.

For total fatty acid analysis, lipid extracts were subjected to hydrolysis by 0.5 M hydrochloric acid in acetonitrile and water (9:l, *v*/*v*) for 45 min at 100 °C [49]. After hydrolysis, fatty acids were recovered in chloroform, dried, and reconstituted in water-saturated butanol and methanol (1:1, *v*/*v*), and analyzed by LC-MS/MS as described above. Fatty acids were detected in negative mode ESI by measuring precursor ion in SIM mode and quantitated by normalizing against deuterated internal standard (Oleic acid-d_17_) that was spiked in before extraction.

#### 4.2.3. LC-MS/MS Analysis

LC-MS/MS analysis of lipid extracts was carried out on a 6495A QQQ mass spectrometer interfaced with an Agilent 1290 series HPLC system (Agilent Technologies, Santa Clara, CA, USA). The dried lipid extracts were reconstituted in 100 µL of methanol and water-saturated butanol (1:1, *v/v*) containing 10 mM of ammonium formate and analyzed using a dynamic MRM approach (dMRM). Mass spectrometry settings and MRM transitions for each lipid class, subclass, and individual species were kept as described previously [48]. A step gradient consisting of solvent B (10 mM ammonium formate in isopropanol/acetonitrile/water (90/10/1, *v*/*v*/*v*) and solvent A (10 mM ammonium formate in water/isopropanol/acetonitrile (50/30/20, *v*/*v*/*v*) was used for separation of the lipid species over a total run time of 15 min. Isolation widths were set to unit resolution for both Q1 and Q2. Blanks and pooled QC samples (prepared by pooling of lipid extracts) were interspersed in the sample sequence to monitor the carryover and reproducibility of the lipidomics data.

#### 4.2.4. Data Analysis and Quantitation

Peak integration was carried out using MassHunter software (B.10; Agilent Technologies). Manual inspection of raw peaks was carried out to ensure correct peak picking, and the peak area data were exported in csv format for further analysis. Lipid species with an analytical coefficient of variation of more than 20% (based on QC) and S/N < 5 were discarded from further analysis. The peak area of each endogenous lipid species was normalized to the corresponding class-specific internal standards for quantitation, as described in Table S2.

#### 4.2.5. Statistical Analysis

Statistical analysis was carried out by using the MetaboAnalyst software suite [50]. Principal component analysis (PCA) was implemented to explain the variance in the data in an unsupervised manner. Univariate analysis was used for Student’s t-test (threshold FDR < 0.05) and fold change (cut-off = 2) estimation, both represented as a volcano plot. Scatter plots for individual concentrations of representative lipid species were plotted in Graphpad Prism (9.1.0). Total fatty acid data were also analyzed in Graphpad Prism by implementing t-tests with a threshold of *p* < 0.05 for significance.

#### 4.2.6. Immunohistochemical Staining for SCD and Scoring of Staining

Following this, 4 µm-thick tissue sections were stained with hematoxylin and eosin for histological evaluation and diagnosis. Expression of SCD was determined on 4 µm-thick tissue sections of OCCC or control ovarian tissue using antibody clone CD.E10 (ThermoFisher Scientific, Singapore) at 1:1000 dilution, a Leica Bond III automated stainer (Leica Biosystems, Melbourne, Victoria, Australia), and Leica Bond DAB polymer detection kit DS 9800 (Leica Microsystems, Singapore, Singapore). The staining intensity was scored by a pathologist (1+ for weak staining, 2+ for moderate staining, 3+ for strong staining), and the h score was calculated in the following manner to take into account the extent as well as intensity of staining: 1 ∗ (% area with 1+ staining intensity) + 2 ∗ (% area with 2+ staining intensity) + 3 *(% area with 3+ staining intensity).

## 5. Conclusions

We have demonstrated the feasibility of using archival FFPE specimens to perform lipidomic analysis to characterize alterations in lipid species in cancer. Using this methodology, we identified differential levels of several lipid species in OCCC compared to the controls, as well as an overall increase in total levels of unsaturated fatty acids. We confirmed the upregulation of stearoyl-CoA desaturase expression in OCCC by immunohistochemistry.

## Data Availability

The data of this study are included in the manuscript and Supplementary Materials. Data and information about reagents/methods are also available from the authors.

## Supplementary Materials

The following are available online at www.mdpi.com/article/10.3390/metabo11090597/s1, Figure S1: Relative distribution of different lipid species represented as percent of the total class levels (A) PC species > 1% and (B) PC species < 1% of the total; (C) PE; (D) PI; (E) SM species > 1% and (F) SM species < 1% of the total, Table S1: Clinical characteristics of the study participants, Table S2: Targeted lipidomics method details, Table S3: Lipid species with a two-fold change between OCCC and control, Table S4: Significant lipid species from two sample *t*-tests with *p*-value (FDR adjusted) < 0.05, Table S5: Details of the lipid species from Volcano plot analysis with at least two-fold change and *p*-value (FDR adjusted) < 0.05, Table S6: Details of fatty acids in individual samples represented as percent of the total concentration, Table S7: IHC scores of OCCC and paired control specimens (*n* = 5), Table S8: Pathway analysis of lipid species that showed differential levels in OCCC as compared to the controls.
