# Supplementary material for: Lipidomic Analysis of Archival Pathology Specimens Identifies Altered Lipid Signatures in Ovarian Clear Cell Carcinoma
Source: Metabolites. 2021 Sep 3;11(9):597. doi: 10.3390/metabo11090597 (PMC8469522; doi:10.3390/metabo11090597)
Supplement: Supplementary file 1 [file metabolites-11-00597-s001.zip › metabolites-1321261-supplementary/Supplementary Figure S1.pdf]

Supplementary Figure S1: Relative distribution of different lipid species represented as percent of the total class levels **(A)** PC species > 1% and **(B)** PC species < 1% of the total; **(C)** PE; **(D)** PI; **(E)** SM species > 1% and **(F)** SM species < 1% of the total

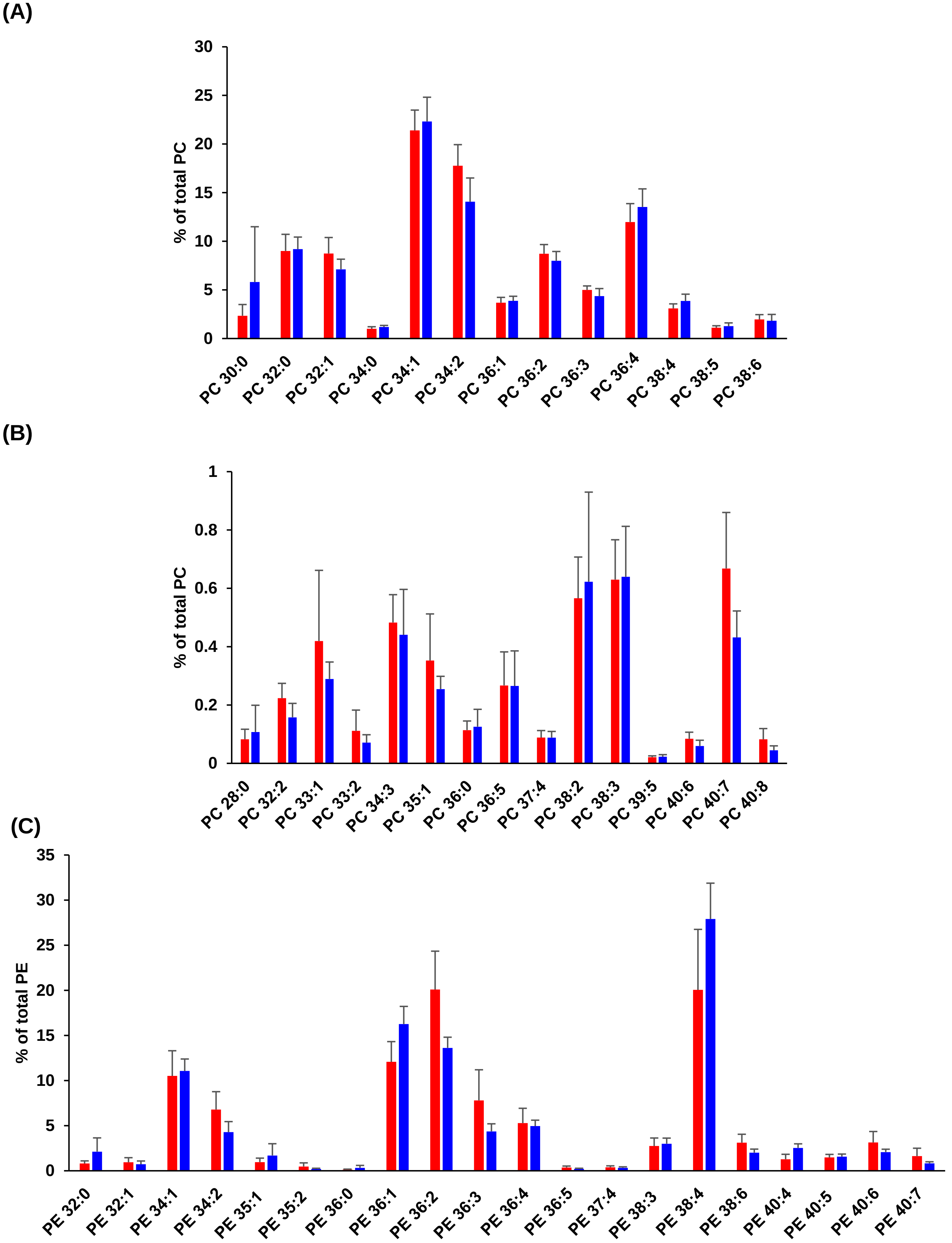
(A)
% of total PC
0
5
10
15
20
25
30
PC 30:0
PC 32:0
PC 32:1
PC 34:0
PC 34:1
PC 34:2
PC 36:1
PC 36:2
PC 36:3
PC 36:4
PC 38:4
PC 38:5
PC 38:6
(B)
% of total PC
0
0.2
0.4
0.6
0.8
1
PC 28:0
PC 32:2
PC 33:1
PC 33:2
PC 34:3
PC 35:1
PC 36:0
PC 36:5
PC 37:4
PC 38:2
PC 38:3
PC 39:5
PC 40:6
PC 40:7
PC 40:8
(C)
% of total PE
0
5
10
15
20
25
30
35
PE 32:0
PE 32:1
PE 34:1
PE 34:2
PE 35:1
PE 35:2
PE 36:0
PE 36:1
PE 36:2
PE 36:3
PE 36:4
PE 36:5
PE 37:4
PE 38:3
PE 38:4
PE 38:6
PE 40:4
PE 40:5
PE 40:6
PE 40:7

(D)

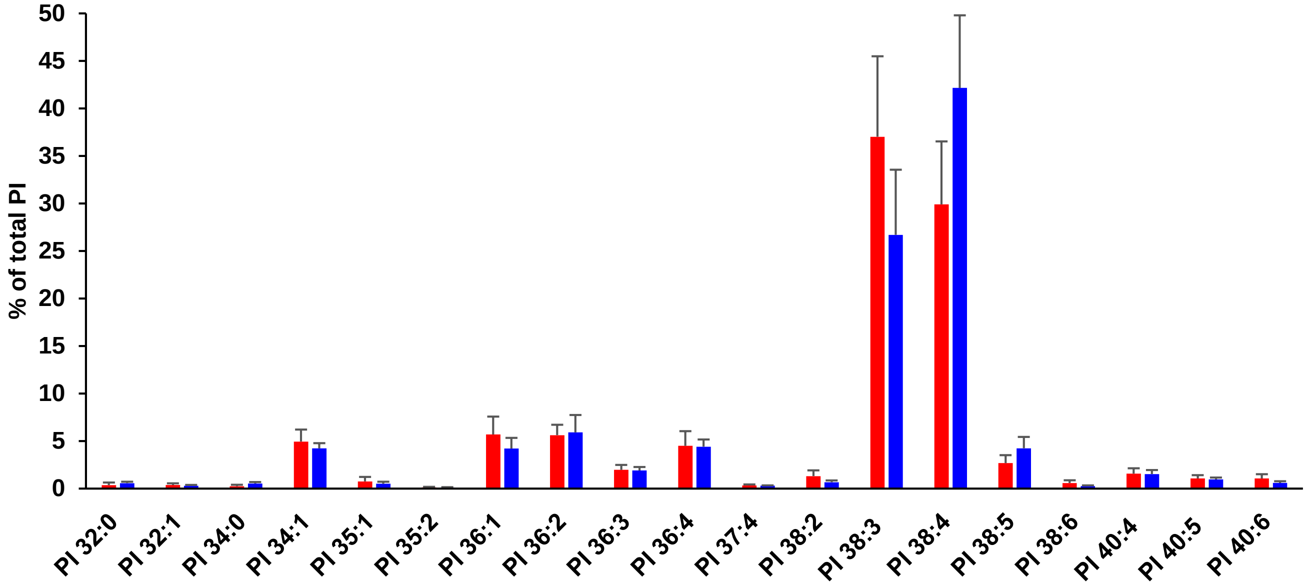
% of total PI
0
5
10
15
20
25
30
35
40
45
50
PI 32:0
PI 32:1
PI 34:0
PI 34:1
PI 35:1
PI 35:2
PI 36:1
PI 36:2
PI 36:3
PI 36:4
PI 37:4
PI 38:2
PI 38:3
PI 38:4
PI 38:5
PI 38:6
PI 40:4
PI 40:5
PI 40:6

(E)

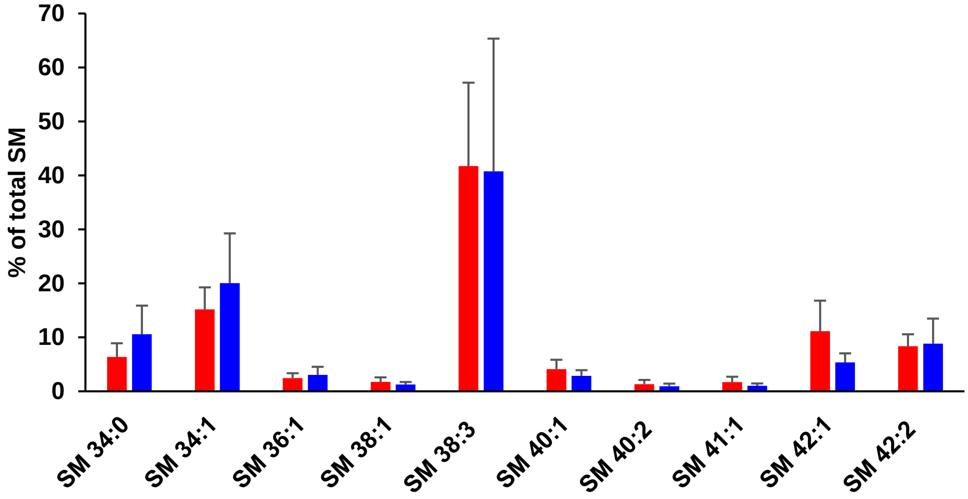
% of total SM
0
10
20
30
40
50
60
70
SM 34:0
SM 34:1
SM 36:1
SM 38:1
SM 38:3
SM 40:1
SM 40:2
SM 41:1
SM 42:1
SM 42:2

(F)

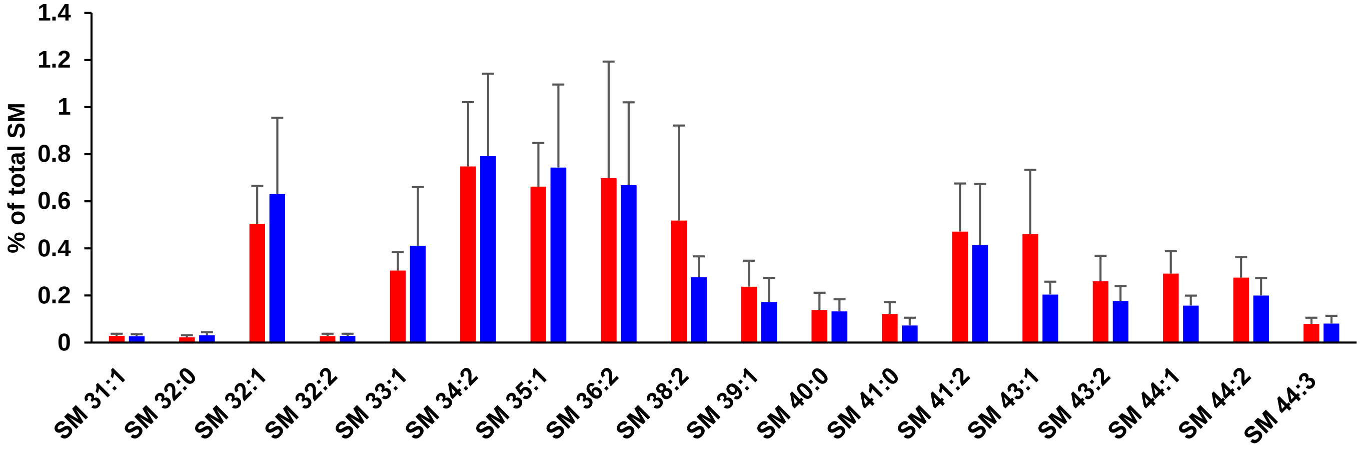
% of total SM
0
0.2
0.4
0.6
0.8
1
1.2
1.4
SM 31:1
SM 32:0
SM 32:1
SM 32:2
SM 33:1
SM 34:2
SM 35:1
SM 36:2
SM 38:2
SM 39:1
SM 40:0
SM 41:0
SM 41:2
SM 43:1
SM 43:2
SM 44:1
SM 44:2
SM 44:3
